# Proceedings of the 17^th^ International Congress of Myriapodology, Krabi, Thailand

**DOI:** 10.3897/zookeys.741.24612

**Published:** 2018-03-07

**Authors:** Pavel Stoev, Gregory Edgecombe D.

**Affiliations:** 1 National Museum of Natural History, BAS, Sofia, Bulgaria; 2 Pensoft Publishers, Sofia, Bulgaria; 3 The Natural History Museum, London, United Kingdom

This special issue of ZooKeys assembles a collection of contemporary research devoted to myriapods presented at the 17^th^ International Congress of Myriapodology, held from 23 to 26 July 2017 in Krabi, Thailand. The congress was organised by Prof. Somsak Panha and his team from the Animal Systematics Research Unit of Chulalongkorn University in Bangkok. This is the third ZooKeys special issue emerging from a myriapodological congress following those of the 15^th^ and 16^th^ congresses in Australia and the Czech Republic, respectively: Mesibov R & Short M (2011) Proceedings of the 15^th^ International Congress of Myriapodology. ZooKeys 156: 139 pp. and Tuf IH & Tajovský K (2015) Proceedings of the 16^th^ International Congress of Myriapodology. ZooKeys 510: 278 pp.

The current issue comprises 13 articles by 35 authors from 10 countries (Austria, Australia, Brazil, China, Czech Republic, Georgia, Germany, Russia, Taiwan, UK). Two articles are devoted to the biogeography of myriapods of the Himalayas and lowland Altai (Golovatch and Martens, Nefedev et al., respectively). Reip and Wesener investigate the haplotype diversity and biogeography of the familiar Black Pill Millipede, *Glomeris marginata*, throughout Europe and draw conclusions on the taxonomic status of a number of subspecies and colour morphs known in this widespread, model millipede species. Kokhia and Golovatch provide an annotated checklist of the millipedes of Georgia.

**Figure 1.**
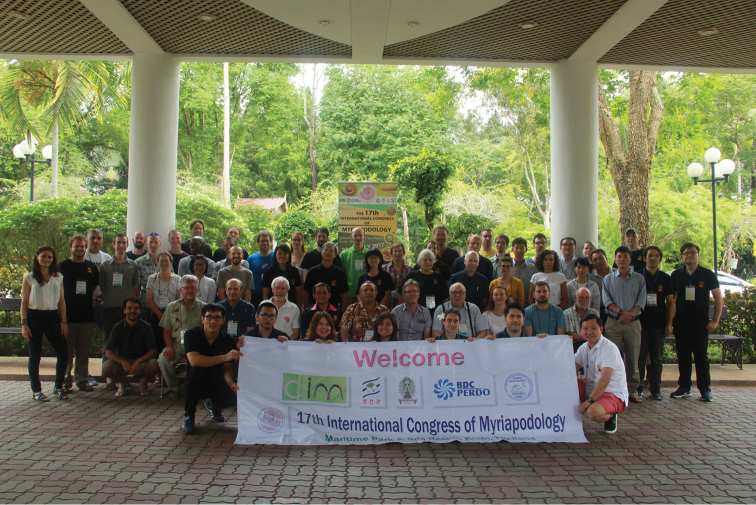
Group photo of the 17^th^ International Congress of Myriapodology.

Two papers focus on anatomical structures of the head capsule and their phylogenetic significance. These involve documenting the tentorium of sphaerotheriid millipedes (Moritz et al.) and the epipharynx and hypopharynx in the centipede genus *Lithobius* Leach, 1814 (Ganske et al.). Another paper deals with the conservation status of Brazilian myriapods based on recent assessments following the IUCN criteria and discusses some practical implications for their conservation (Karam-Gemael et al.). One contribution (Decker et al.) describes the online platform VIRMISCO (Virtual Microscope Slide Collection) – a digital archive for microscope slides that enables users to view, search, rotate, zoom, measure, etc., important type objects.

Five papers in this special issue are devoted to systematic description of altogether nine new myriapod species from East Asia and Australia, these belonging to the centipede genus *Lithobius* (Chao et al., Ma et al., Pei et al.), and the millipede genera *Lophoturus* (Huynh and Veenstra) and *Glyphiulus* (Jiang et al.).

We are grateful to the referees of contributions to this issue for careful and prompt work that improved the quality of the accepted manuscripts.

We look forward to the next International Congress of Myriapodology, which will be held in 2019 and hosted by Prof. Zoltan Korsós and his team at the Hungarian Natural History Museum.

Pavel Stoev, Gregory D. Edgecombe

